# Developmental trajectories of paediatric headache – sex-specific analyses and predictors

**DOI:** 10.1186/s10194-016-0627-8

**Published:** 2016-04-14

**Authors:** Corinna Isensee, Carolin Fernandez Castelao, Birgit Kröner-Herwig

**Affiliations:** Georg-Elias-Müller-Institute of Psychology, Department of Clinical Psychology and Psychotherapy, University of Göttingen, Gosslerstraße 14, 37073 Göttingen, Germany; Department of Child and Adolescent Psychiatry and Psychotherapy, University Medical Center of Göttingen, Göttingen, Germany

**Keywords:** Paediatric headache, Developmental trajectories, LCGA, Sex-specific analyses

## Abstract

**Background:**

Headache is the most common pain disorder in children and adolescents and is associated with diverse dysfunctions and psychological symptoms. Several studies evidenced sex-specific differences in headache frequency. Until now no study exists that examined sex-specific patterns of change in paediatric headache across time and included pain-related somatic and (socio-)psychological predictors.

**Method:**

Latent Class Growth Analysis (LCGA) was used in order to identify different trajectory classes of headache across four annual time points in a population-based sample (*n* = 3 227; mean age 11.34 years; 51.2 % girls). In multinomial logistic regression analyses the influence of several predictors on the class membership was examined.

**Results:**

For girls, a four-class model was identified as the best fitting model. While the majority of girls reported no (30.5 %) or moderate headache frequencies (32.5 %) across time, one class with a high level of headache days (20.8 %) and a class with an increasing headache frequency across time (16.2 %) were identified. For boys a two class model with a ‘no headache class’ (48.6 %) and ‘moderate headache class’ (51.4 %) showed the best model fit. Regarding logistic regression analyses, migraine and parental headache proved to be stable predictors across sexes. Depression/anxiety was a significant predictor for all pain classes in girls. Life events, dysfunctional stress coping and school burden were also able to differentiate at least between some classes in both sexes.

**Conclusions:**

The identified trajectories reflect sex-specific differences in paediatric headache, as seen in the number and type of classes extracted. The documented risk factors can deliver ideas for preventive actions and considerations for treatment programmes.

## Background

The aim of the current study was to identify developmental courses of headache in a longitudinal study including a population-based sample of children and adolescents aged between 9 and 14 years at first assessment. Besides the analysis of trajectories that differ significantly with regard to their starting level and average growth, we also wanted to identify predictors that influence the class membership of the individuals.

Headache is the most common pain disorder in children and adolescents [1, 2]. In an epidemiological study by van Gessel, Gaßmann and Kröner-Herwig [3] children between 9 and 14 years reported a six-month prevalence that ranged from 56.6 to 75.1 %, depending on the age and sex of the child. A systematic review of population-based studies found an overall prevalence rate of 58.4 % (95 % CI = 58.1–58.8 %) in children and adolescents younger than 20 years [4].

Various studies indicate sex differences in headache prevalence, with girls suffering more often from headache than boys [5, 6]. However, this sex discrepancy seems to become significant only from adolescence on [7, 8].

Among different primary headache types, migraine and tension-type headache are most common in childhood and adolescence, with the latter being most frequent [2]. In the study by van Gessel et al. [3] 17.6 % of the examined children were diagnosed with a tension-type headache, whereas only 13.1 % reported to suffer from migraine.

### Correlates of headache

Headache cannot be understood in terms of a pure somatic phenomenon, but is associated with diverse dysfunctions and psychological symptoms [9, 10]. Especially internalising symptoms like depression and anxiety were repeatedly found to be associated with headache [11–13]. In a study by Stanford, Chambers, Biesanz & Chen [14], a higher level of depressive symptoms at first panel was associated with a higher headache frequency over the course of the assessment.

Also, various studies found a positive association between headache and dysfunctional stress coping [15, 16]. In a study by Saile and Scalla [17], children with chronic headache showed more dysfunctional stress coping strategies (rumination, resignation, aggression) and reported less positive self-instructions compared to children without headache.

Moreover, genetic factors were repeatedly discussed in the context of paediatric headache [18, 19]. In a study by Kröner-Herwig and Gaßmann [20] an almost four times higher risk of suffering from migraine was found when one parent suffered from migraine as well. For tension-type headache the authors found an Odds Ratio (OR) of 2.50.

Additionally, studies indicate that being diagnosed with migraine at baseline may influence the course of headache and the headache frequency at a later time point. Several studies report different remission rates. Whereas, in the study by Kienbacher et al. [21] only 26 % of patients diagnosed with migraine at baseline were headache-free at follow-up (6.6 +/− 1.6 years after baseline), Guidetti and Galli [22] found a percentage of 51 % headache-free children after six years.

Besides psychological and genetic factors, several cross-sectional and prospective studies indicate that the family climate, school burden, as well as the number of life events also increase the probability of developing and maintaining headache [23–29].

Other studies point to the risk of developing a chronic state of headache [30, 31]. Bille [32] showed that 53 % of children reporting migraine at first assessment (between 7 and 13 years old) still suffered from it after 30 years. This underlines the relevance of systematically examining the development of headache across childhood and adolescence and identifying predictors for favourable and unfavourable developmental courses of headache in order to analyse the determinates of differences in individual development.

### Previous research on paediatric headache and longitudinal studies

The majority of longitudinal studies concerning paediatric headache only analysed two assessment points, with the aim of identifying predictors for headache at a single later date. However, these kinds of studies do not detect trajectories over time and cannot describe intraindividual and interindividual changes in development.

Until now, only few studies focused on the description of paediatric headache courses over more than two assessment points with appropriate statistical methods.

Stanford et al. [14] utilised structural equation modelling to identify trajectories for headache, abdominal, and back pain in Canadian adolescents between 10 and 11 years at first assessment (*n* = 2 488). The assessment was conducted every two years over a 10-year period. Results showed increasing headache frequencies over time. Moreover, the mother’s headache as well as the child’s depression/anxiety were predictors for high headache prevalence over all assessment points. Again, the finding of higher headache prevalence over the whole of adolescence in girls compared to boys was confirmed. However, with the selected statistical method the authors were only able to identify one single trajectory for every pain type and could not consider the potential heterogeneity in the population.

In the study by Dunn, Jordan, Mancl, Drangsholt and Resche [33], different trajectory clusters of facial, back, and abdominal pain as well as headache in adolescents between 11 and 14 years (*n* = 1 136) were examined. The data were collected every three months over three years in a population-based sample. Pain-related psychological and somatic variables were included as predictors in order to identify potential differences between the classes of a pain type. The authors utilised a subtype of Growth Mixture Modeling (GMM), the Latent Class Growth Analysis (LCGA). It assumes that there is no within class variance among individuals of one class so that the variances of the growth factors are fixed at zero [34]. The research group of Dunn [33] could identify four different trajectories for headache. Children and adolescents assigned to the trajectory class that was characterised by a high probability of headache over the whole assessment time, showed higher scores of depression and somatisation and the lowest life satisfaction at first assessment compared to children and adolescents of cluster one to three. Other potentially relevant factors associated with headache like genetic or family-related factors, the specific headache type (migraine vs. tension-type headache), the level of anxiety, school burden or the number of life events were not included in the analyses. Moreover, sex-specific analyses were not carried out.

A study that analysed adults (mean age = 41.8 years, *SD* = 14.61) focused on migraine as a pain type [35]. In a ten-year follow-up study (with eight assessment points), the authors investigated the number of trajectory classes concerning the severity, frequency and duration of migraine. The examined adults were outpatients with a diagnosis of migraine (*n* = 1 048). The authors used GMM as the statistical method. Results showed that for the headache frequency three different trajectories, i.e., classes could be identified (low, medium, high). In spite of previous results concerning associations between migraine and other somatic dysfunctions as well as psychological symptoms, the authors only included co-morbid symptoms of migraine like e.g., vomiting, aura, and nausea as potentially influencing the trajectories. Furthermore, they did not execute sex-specific analyses, which however seem important in the analysis of headache trajectories over time [8]. Although, this study focused on adults and not children, the methods and analytic procedures used are very similar to the current study and present a working model for our work.

In summary, the data base concerning trajectories of headache in childhood and adolescence is very limited. First, only two studies are known of that included the interesting sample of children and adolescents. However, only one of these studies was able to consider the possible heterogeneity in the population by using the adequate statistical method (LCGA) when analysing longitudinal data [35, 36]. Second, prior studies disregarded the important role of risk factors when examining developmental courses of paediatric headache. Third, an analysis of possible sex differences concerning patterns of change in paediatric headache was not considered, although a very convincing data base exists that reports gender effects in the prevalence of headache.

### Methodological considerations: Latent Class Growth Analysis (LCGA)

LCGA allows the consideration of unobserved heterogeneity in trajectories and permits the analysis of multiple developmental courses in a given population. Whereas conventional growth models (Growth Curve Modeling, e.g., Structural Equation Modeling) [37] consider a single (mean) trajectory as sufficient to represent the whole population, LCGA permit variations of trajectories in longitudinal data, leading to the description of latent classes that differ in terms of their intercept (initial level), as well as their slope (average growth) [34]. As a result, individuals are assigned to distinct subgroups. In LCGA, variances of the growth parameters are fixed at zero and are not randomly estimated. In consequence, there is no covariance between intercept and slope and thus there are fewer parameters to estimate. This can avoid unstable solutions and problems in model convergence [34, 38].

In order to determine the appropriate number of classes/ subgroups, i.e., to find the best model for the examined data, analyses with different numbers of classes are carried out, and fit indices are used to support the decision on the optimal model. Besides *Bayes’ Information Criterion* (BIC) and *Akaike’s Information Criterion* (AIC), also *Entropy* as well as the *Posterior Class Probability* can be used to extract the correct number of classes [35]. Concerning *BIC* and *AIC*, lower values indicate a better model fit. In contrast, for *Entropy*, ranging from 0.00 to 1.00, higher values indicate a better fit. The *Posterior Class Probability* ranges from 0.00 to 1.00 and refers to the probability with which a child is correctly classified in one class as compared to the other classes. Here again, higher scores indicate good categorisation. Another criterion to support the extraction process is the *Bootstrapped Likelihood Ratio Test* (*BLRT*). This test allows the comparison of a k class model with a k-1 class model. Small *p* values (<0.05) indicate the rejection of a k-1 model [36, 39].

Besides statistical criteria supporting the decision on the best fitting model, content-related considerations and theoretical implications must also guide the decision process [40, 41]. The usefulness of the latent classes must have face validity. For example, classes must be distinguishable from each other and of sufficient size, otherwise the stability and reliability of the estimates is questionable [42]. Additionally, the added class should prove to provide results that add to the understanding and meaning of the data [43]. Moreover, prior studies with comparable populations, research questions and methodical preconditions should be referred to as indicators of validity.

In order to support the identification of different classes in LGCA, predictors that are expected to correlate with the outcome variable can be included in the model. It is assumed that these predictors exert influence on the class membership.

### Hypotheses

In the current study German children and adolescents of a population-based sample were analysed regarding their self-reported headache frequency over four annual assessment points. Headache frequency was assessed by the number of headache days in the last six months. This is the first study to describe sex-specific courses of headache over time, including genetic, psychosocial and family related predictors in a sample of children and adolescents (9 and 14 years) at first assessment. Since paediatric headache can be associated with functional impairment and a lower quality of life, including psychological and somatic aspects [3, 44, 45], the identification of risk factors as well as factors maintaining unfavourable courses of headache is of high interest, especially regarding the potential of preventive action and considerations for the indication of treatment.

Taking account of the convincing data bases on sex-specific differences in headache prevalence and headache frequency, we conducted separate analyses for boys and girls. We expected to find different trajectories of headache in boys and girls enabling us to describe differences in courses across these classes.

Due to the deficient evidence on sex-specific courses of headache, no concrete hypotheses concerning the exact number of classes for boys and girls could be inferred. However, we did not expect more than four classes for both sexes, respectively.

As a population-based sample was analysed, one large class with no or a very low headache frequency over the whole assessment time, for both boys and girls was expected. This class would be larger in the boys’ subsample.

Moreover, a class with a high level of headache across time, in both boys and girls should be identified. This class would be larger in the subsample of girls and children belonging to this class would already have a moderate to high headache frequency at the first assessment.

Concerning the trajectories of the other classes, we expected that they would be defined by a low to moderate number of headache days across all four assessment points, with small changes in slope across time.

Furthermore, various headache related predictors that are associated with headache prevalence and its maintenance [11, 12, 17, 23, 24] were used in order to evaluate their influence on the class membership. The diagnosis of migraine, parental headache, internalising symptoms, dysfunctional stress coping, stressful life events, school burden, as well as a negative family climate at wave one were expected to significantly increase the risk of belonging to the highly or at least moderately affected headache groups.

## Methods

### Participants and procedure

The study sample was drawn from a large longitudinal population study. At first assessment in 2003, 8800 families (with children from 7 to 14 years) were randomly drawn from community directories in Southern Lower Saxony and the city of Hannover (Germany) and were asked to report on headache and other relevant psychological variables via postal surveys. For this wave we received a response rate of 63.5 % (*n* = 5586). These families were again contacted for the three following annual panels (2004, 2005, 2006). For every wave, participating children and parents received separate questionnaires and were informed about the anonymous analysis of the data. Due to difficulties of young children in responding to the questions, only children of nine years and older received a child questionnaire [46].

For the current study, data from all four waves of this large population study were included. For our analyses we first had to exclude families with children younger than 9 years since we drew on the self-reported headache frequency of the questioned children. Secondly, for our study sample, we had to exclude cases with more than 50 % missing data. These two steps led to a reduction of the sample size (*n* = 3985). Additionally, for our longitudinal analyses we only included those children that reported on their headache frequency in at least two of the four waves, resulting in a further reduction (*n* = 3227), with a relatively equal distribution of boys (48.8 %) and girls (51.2 %). For our final study sample, including children of all four waves, the mean age at wave one was 11.34 years (*SD* = 1.71; boys: *M* = 11.35, *SD* = 1.68; girls: *M* = 11.33, *SD* = 1.74). Concerning socioeconomic status 45.7 % belonged to the middle and 38.1 % to the upper status group [47].

The ethics committee of the German Association of Psychology approved the study protocol [44]. More details about the study procedure can be found in Kröner-Herwig et al. [44].

### Measures

#### Outcome variable: number of headache days

In order to assess paediatric headache, children were asked at all four waves if they had experienced headache in the last six months at least once a week, at least once a month or less than once a month. Depending on their answer they were asked to specify the frequency of experienced headache in the chosen period. This information was extrapolated with corresponding loading for the last six months, so that the variable could reach values between 0 and 182. Higher values indicated more days of headache in the last six months. The outcome variable relates to all headaches and is not limited to migraine. It focuses on headache frequency in general.

### Predictors

#### Migraine

In the interest of diagnosing migraine, our questions concerning the child’s headache symptoms were defined in accordance with the International Classification of Headache Disorders, 2nd edition (ICHD-II) [48]. An algorithm was used to categorise the children based on their self-report. This procedure was validated in prior studies [49, 50] and can be considered as sufficiently adequate in diagnosing headache types in children and adolescents. We built a binary variable to assess migraine at the first wave (‘no migraine’ (0), ‘migraine’ (1)).

#### Parental headache

At the first assessment, the parent (in 75.2 % of cases mothers) reported on his/her own headache frequency in the last six months and was also questioned about the partner’s headache. A four category frequency variable for both parents each (‘no headache’ (0), ‘less than monthly’ (1), ‘at least monthly’ (2), ‘at least weekly’ (3)) was used. Again, the rank data were transformed into a dichotomous variable for maternal and paternal headache, respectively, (‘no recurrent headache’ (0), ‘recurrent headache’ (1)). To facilitate later analyses, we decided to include the information of both parents’ headache into one single variable (‘no headache in either parent’ (0), ‘headache in one or both parents’ (1)).

#### Depression/anxiety

Depression/anxiety, often denoted as ‘Internalising Symptoms’, were assessed by eight selected items from the Youth Self Report at the first assessment point (YSR; example: ‘I feel guilty’) [51], referring to the last three months. In order to achieve a better comparability with other scales of the questionnaire, the originally three-point-scale was transformed into a five-point scale (‘never’ (1) to ‘always’ (5)). The items were selected on the basis of strong item-scale correlations in the original YSR [52]. Higher scores indicated a higher level of internalising symptoms. The shortened scale showed a good homogeneity (Cronbach’s *α* = 0.84) and correlated highly with the comprehensive scale (*r* > 0.80) [52].

#### Stressful life events

The occurrence of critical life events was assessed according to the procedure of Kröner-Herwig and Gaßmann [20]. We utilised the parental report of eight critical events in the child’s life (loss of a family member, financial burden, new person in the family, chronic illness or accident in the family, nursing of a family member, change of school, attendance at boarding school) at the first wave. The items were extracted from the Mannheimer Parent Interview (MEI) [53] and referred to stressful events in the last five years before the assessment. The sum of the eight events with scores ranging from 0 to 8 was used as the predictor variable. Higher scores indicated more life events. According to Esser et al. [53] content and face validity can be expected.

#### Dysfunctional stress coping

Dysfunctional Stress Coping was assessed at the first wave by five items from the Stress Coping Inventory [54] (example: ‘When I am under stress…. I tent to pretend I am sick’), with a five-point rating scale from ‘never’ (1) to ‘always’ (5). The items were chosen with regards to strong correlation with the comprehensive scale (0.74 ≤ *r* ≤ 0.96) [55, 56]. The mean of the scale was used for every child, higher scores indicated a more dysfunctional stress coping. The shortened scale showed a good homogeneity (Cronbach’s *α* = 0.76) [20].

#### School burden

Questions assessing potential school burden were conceptualised according to the studies of Anttila, Metsähonkala, Helenius and Sillanpää [57] as well as Karawautz and colleagues [58]. The children had to report on eight items including conflict with peers and a poor performance in school (example: ‘I get bullied or tormented by peers at my school’), on a five-point-rating scale from ‘never’ (1) to ‘always’ (5). The mean of the items was used for every child (Cronbach’s *α* = 0.67). A higher score indicated a higher school burden. The variable was assessed at the first wave.

#### Negative family climate

The family climate was measured by three items, derived and adapted from the Mannheimer Parent Interview at the first time point (MEI) [53]. The items assessed the child’s satisfaction concerning the amount and kind of family activities and the perceived possibility to talk about conflicts within the family (‘Do you have enough time for each other?’; ‘Do you like the activities your parents are doing with you?’; ‘Do you talk about your sorrows and problems with your family members?’) with a five-point scale from ‘never’ (1) to ‘always’ (5)). For the current study the mean score was used for every child. Higher scores indicated a more negative climate. Cronbach’s alpha was satisfactory (Cronbach’s *α* = 0.66).

### Statistical analyses

In accordance with the recommendation of Muthén [59], a conditional model was used, including the predictors in our analyses, trying to analyse their influence on the class membership (multinomial logistic regression) and to identify the best fitting model for the data.

When estimating models with LCGA in Mplus (Mplus 6.1 software) [60], the algorithm of robust maximum likelihood estimation (MLR) is used. This technique is appropriate for non-normal data. In order to estimate missing data, Mplus uses full information maximum likelihood (FIML) for the dependent variables. However, this procedure cannot be used for the predictors [60]. This led to a reduced sample size for the multinomial logistic regression analyses. However, because of the high covariance coverage (ranging from 0.68 to 1.00) we still received reliable and valid model estimation.

To avoid local maxima, the estimations were carried out with at least 400 random sets of starts (one-class solution) and were increased for more classes accordingly. For model selection, we used the *BIC*, values of *Entropy* and *Posterior Class Probability*, the *BLRT* as well as theoretical implications.

## Results

### Headache frequency and predictors: differences in sex and time

On average, the analysed total sample showed a rather low number of headache days in the last six months, in all four waves. Means ranged from *M* = 13.21 (*SD* = 24.92) at first assessment to *M* = 14.96 (*SD* = 26.02) at the fourth period. A significant increase in headache frequency from wave one to two (*p* = 0.004; *d* = 0.06) was observed, a slight and insignificant decrease in wave three (*p* = 0.073) was found as well as another significant enhancement in wave four (*p* = 0.010; *d* = 0.06).

Across all four time points, significant sex differences were seen, with girls showing higher headache frequencies across all four waves (*p* < 0.001; Table 1). In addition, the girls’ subsample showed a significant increase of headache frequency from wave one to two (*p* < 0.001; *d* = 0.12), with an insignificant decrease in wave three (*p* = 0.104) and another significant increase in wave four (*p* = 0.004; *d* = 0.45). In contrast, for the boys’ subsample no significant changes of headache frequency across time were identified (all *p* > 0.05).

**Table 1:** Descriptive statistics of outcome variables and predictors

	Girls			Boys		
Variable	M (SD) / n	Range	N	M (SD) / n	Range	N
Head1	15.45 (27.27)	0–182	1640	10.84 (21.97)	0–182	1560
Head2	18.37 (30.79)	0–182	1477	10.23 (20.44)	0–182	1429
Head3	16.92 (27.70)	0–182	1311	10.27 (20.77)	0–182	1267
Head4	19.11 (29.41)	0–182	1228	10.50 (20.87)	0–182	1158
Mig	182^a^	yes/no	1640	137^a^	yes/no	1560
Par	679^b^	yes/no	1393	671^b^	yes/no	1326
Dep/Anx	1.74 (0.62)	1.00–4.50	1607	1.55 (0.51)	1.00–4.83	1523
LE	0.94 (1.11)	0.00–6.00	1583	0.96 (1.14)	0.00–6.00	1527
Cop	2.22 (0.75)	1.00–4.80	1604	1.99 (0.65)	1.00–5.00	1522
School	1.67 (0.44)	1.00–4.20	1643	1.68 (0.45)	1.00–4.00	1567
Fam	2.21 (0.80)	1.00–5.00	1625	2.30 (0.78)	1.00–5.00	1543

*Head1-4* days of headache in the last six months for wave 1–4, *Mig* migraine, *Par* parental headache, *Dep/Anx* depressive/ anxiety symptoms, *LE* life events, *Cop* dysfunctional stress coping, *School* school burden, *Fam* negative family climate

^a^number of children in subgroup reporting to have migraine

^b^number of children in subgroup reporting to have parental headache in the family

Moreover, at first assessment, girls and boys differed significantly with respect to their level of depressive/anxiety symptoms (*p* < 0.001; *d* = 0.33), their stress coping (*p* < 0.001; *d* = 0.33) as well as the perception of the family climate (*p* = 0.002; *d* = 0.10). Moreover, more girls reported to suffer from migraine at wave one than boys (*p* = 0.029, *d* = 0.08). No significant sex differences in the children’s school burden, the number of life events, or the parent’s headache reports (all *p* > 0.05) were identified.

### Conditional model for girls

For the subsample of the girls, we identified the four-class solution as the best fitting model. The *BIC* decreased until a four-class model and increased again with a five-class solution (*BIC*_*1*_ = 74369.548; *BIC*_*2*_ = 36233.008; *BIC*_*3*_ = 34360.569; *BIC*_*4*_ = 33965.944; *BIC*_*5*_ = 34017.236). The classification accuracy proved to be satisfying (*Entropy* = 0.863) with *Posterior Class Probabilities* ranging from 0.86 to 0.98. A significant value of the *BLRT* (*p* < 0.001) indicated that the four-class model was favoured as compared to the three-class solution. Including a quadratic growth factor improved the model fit. Stable means of growth factors as well as the same distributions and class sizes across different starting values indicated a stable and reliable solution.

**Fig. 1:**
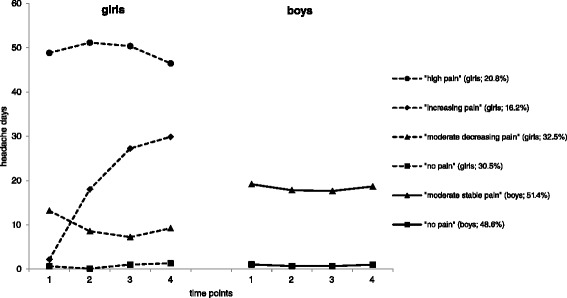
Trajectories for girls (*n* = 1298) and boys (*n* = 1236)

The largest class in the girls’ subsample (32.5 %; Fig. 1) showed a moderate headache frequency at the starting level. Also, a significant decreasing trend that accelerated over time (*s* = − 6.270, *p* < 0.001; *q* = 1.651, *p* < 0.001; class 3: ‘*moderate decreasing pain class*’) was identified. The second largest class (30.5 %) displayed a very low level of mean headache days across all four waves, without significant changes in slope (*s* = 0.148, *p* = 0.287; *q* = 0.025, *p* = 0.581; class 4: ‘*no pain class*’). The third group of girls (20.8 %) started with a rather high level of headache as compared to all other classes without significant changes of headache days across time (*s* = 3.882, *p* = 0.386; *q* = − 1.557, *p* = 0.262; class 1: ‘*high pain class*’). With regard to the possible range of the outcome variable (0–182), the mean level of headache days of this class cannot be classified as ‘high’. However, in relation to the mean number of headache days of the other classes, this class clearly differs concerning the level of headache frequency and is therefore named as ‘high pain class’. The fourth class of girls (16.2 %) was defined by a low headache frequency at first wave, with a significant acceleration that stabilised by the time of the fourth wave (*s* = 19.143, *p* < 0.001, *q* = − 3.303, *p* = 0.005; class 2: ‘*increasing pain class*’).

For the multinomial logistic regression analyses, we used the ‘no pain class’ as reference class (Table 2). Results revealed that migraine (*OR* = 6.59), parental headache (*OR* = 1.78) as well as depressive/anxiety symptoms (*OR* = 2.12) significantly increased the risk of belonging to the ‘moderate decreasing pain class’ as compared to the class without any pain across time. Concerning the ‘high pain class’ migraine (*OR* = 10.39), parental headache (*OR* = 2.67), depressive/anxiety symptoms (*OR* = 2.72), life events (*OR* = 1.27) and dysfunctional stress coping (*OR* = 1.41) discriminated between this class and the reference class. For the ‘increasing pain class’, depressive/anxiety symptoms (*OR* = 1.73) as well as school burden (*OR* = 1.74) were significantly associated with a higher risk of belonging to this class as compared to the ‘no pain class’.

**Table 2:** Results from multinomial logistic regression analyses for girls (*n* = 1298) and boys (*n* = 1236)

Girls: odds ratios (95 % CI)							Boys: odds ratios (95 % CI)	
Predictors	no vs. moderate decreasing pain		no vs. high pain		no vs. increasing pain		no vs. moderate stable pain	
Mig	6.59***	(2.98–14.57)	10.39***	(4.59–23.48)	1.85	(0.58–5.94)	6.73***	(3.45–13.08)
Par	1.78***	(1.30–2.44)	2.67***	(1.84–3.86)	1.36	(0.91–2.03)	1.80***	(1.40–2.32)
Dep/Anx	2.12***	(1.40–3.21)	2.72***	(1.76–4.22)	1.73*	(1.05–2.85)	1.12	(0.80–1.57)
LE	1.01	(0.86–1.18)	1.27**	(1.07–1.51)	1.13	(0.93–1.36)	1.12*	(1.02–1.26)
Cop	1.12	(0.83–1.52)	1.41*	(1.00–1.98)	1.00	(0.69–1.46)	1.58**	(1.22–2.05)
School	1.01	(0.66–1.55)	1.35	(0.85–2.17)	1.74*	(1.02–2.96)	1.22	(0.89–1.66)
Fam	0.93	(0.75–1.16)	1.29	(0.99–1.68)	1.07	(0.82–1.40)	0.96	(0.81–1.13)

*Mig* migraine, *Par* parental headache, *Dep/Anx* depressive/ anxiety symptoms, *LE* life events, *Cop* dysfunctional stress coping, *school* school burden, *Fam* negative family climate, *CI* confidence interval

*p < 0.05; ** *p* < 0.01; *** *p* < 0.001

### Conditional model for boys

In the boys’ subsample, the *BIC* decreased from the one-class to the two-class solution (*BIC*_*1*_ = 66528.129; *BIC*_*2*_ = 30048.525). Adding a quadratic slope led to an improvement of the model fit. The two-class model showed very good classification accuracy (*Entropy* = 0.964; *Posterior Class Probability* = 0.994–0.987). The *BLRT* also significantly preferred the two-class solution over the one-class model (*p* < 0.001). Variation of starting values led to the same results for class proportions and patterns of change which indicated a stable and reliable model.

In contrast to this, varying starting values in the three-class model led to differing *BIC*s and changes in class distributions and class sizes. The model results indicated convergence problems and lower values for model fit indicators as compared to the two-class model. Therefore, we chose the two-class solution as best fitting model for the boys’ subsample.

The larger class in the boys’ subsample (51.4 %) showed a moderate level of headache at the first wave without any significant changes across time (class 1: ‘*moderate stable pain class*’; *s* = − 1.955, *p* = 0.167; *q* = 0.596, *p* = 0.184; Fig. 1).

Boys of the second class (48.6 %) showed a very low starting level, a significant decrease (*s* = − 0.537, *p* < 0.001) and another significant acceleration from wave three to four (*q* = 0.174, *p* < 0.001). Although we found an increase in headache days, the highest estimated mean value of days was 1.05 at wave four. Therefore, we categorised this class as the ‘*no pain class*’.

Concerning multinomial logistic regression analyses, we again used the ‘no pain class’ as our reference class (Table 2). For the boys’ subsample, migraine (*OR* = 6.73), parental headache (*OR* = 1.80) as well as life events (*OR* = 1.12) and dysfunctional stress coping (*OR* = 1.58) increased the risk of belonging to the ‘moderate stable pain class’ as compared to the ‘no pain’ class.

## Discussion

The present study was conducted with the aim of identifying sex-specific trajectories of paediatric headache in a longitudinal study with a population-based sample. The statistical method of LCGA was used in order to classify children according to their initial headache level and their pathways across time. The identification of genetic, somatic and (socio-)psychological predictors can be the first step for planning effective preventive actions and considering possible treatment programmes. This is the first study to conduct sex-specific longitudinal analyses in a paediatric headache sample of children and adolescents (9 to 14 years old at first wave) using this kind of method and a combination of predictors.

### Results from LCGA

Concordant to our hypotheses we did not find more than four classes for either subsample. For girls a four-class solution was identified. This is in line with Dunn et al. [33]. Here also four classes were extracted.

In accordance with our hypotheses, one class was found with girls reporting no headache across all four assessment points (‘no pain class’; 30.5 %). The ‘moderate decreasing pain class’ (32.5 %) was comprised of girls showing moderate to rather low headache frequencies across time, with significant changes. Post-hoc tests revealed that the majority of girls belonging to the ‘no pain class’ and the ‘moderate decreasing pain class’ were not disabled by headache in their daily or school activities and were able to pursue their school or occupational tasks. This result corresponds to comparable prior studies that found no or a low incidence of paediatric headache in the majority of children of population-based samples [33, 56, 61].

Consistent with our hypotheses, we also identified one group of girls with a high headache frequency at first wave and across all other time points (‘high pain class’; 20.8 %). In post-hoc analyses, we carried out several t-tests to compare the headache-related functional disability between the extracted classes at wave four. As compared to the ‘no pain class’ and the ‘moderate decreasing pain class’ girls of the ‘high pain class’ showed significantly higher impairments in daily and school activities due to headache (all *p* < 0.001).

The ‘increasing pain class’ (16.2 %) was defined by a very low starting level and a significant increase across time. In this respect this class differed from the other classes with rather stable trajectories and only slight changes. As compared to the ‘no pain class’ and the ‘moderate decreasing pain class’ this group of girls reported significantly higher impairments in daily (all *p* < 0.05) and school activities (all *p* < 0.001) and suffered from significantly more disability days due to headache (all *p* < 0.01).

Whereas for most of the cases belonging to the ‘no pain class’ and ‘moderate decreasing pain class’, preventive or therapeutic interventions would probably not be necessary, girls of the ‘increasing pain class’ and ‘high pain class’ seem to be in special need of attention.

In the boys’ subsample, a two-class solution was identified as best-fitting model. In accordance with our hypotheses the ‘no pain class’ (48.6 %) included boys that reported to have had no headache across all time points. In this way, this class is comparable to the ‘no pain class’ of the girls’ subsample. Additionally, we found one class with a moderate headache frequency and significant but small changes across time (‘moderate stable pain class’; 51.4 %). Post-hoc analyses showed that the majority of boys of both classes negated headache-related disability concerning school or social activities at wave four. Referring to the observed time period of four years, there seems to be no need of involving boys from the current study in preventive programs.

The number as well as the patterns of change of the extracted classes in boys and girls reflect sex-specific differences in prevalence of headache and associated pain-related disability between boys and girls. These sex-discrepancies are in accordance with many prior findings [5, 62].

### Results from multinomial logistic regression analyses

In the girls’ subsample, migraine, parental headache and depression/anxiety were significant predictors for the categorisation in the ‘moderate decreasing pain class’ and ‘high pain class’ as compared to the ‘no pain class’. The relevance of these three predictors in the development and maintenance of headache was repeatedly confirmed in several prior studies [21, 63, 64]. Hence, it seems a stable and reliable finding. Furthermore, several studies showed that being diagnosed with migraine is in general associated with diverse somatic, psychological, behavioural and social impairments [11, 63, 65, 66].

Moreover, prior studies reported the importance of parental headache in the development and maintenance of paediatric headache [23, 67]. Besides a familial transfer of a genetic disposition for headache, pain-associated learning mechanisms have repeatedly been discussed [68]. It is assumed that pain-related modelling, i.e., the parental perception of and coping with pain, influence the child’s cognitive, emotional, and behavioural coping with pain and thus can exert an influence on the child’s pain intensity, frequency, and pain-related disability [68].

Furthermore, the probability of belonging to the ‘moderate decreasing pain class’, the ‘high pain class’ and the ‘increasing pain class’ as compared to the ‘no pain class’ was significantly elevated for girls that showed depressive/anxiety symptoms at the first wave. It can be assumed that the reported depressiveness/anxiety is associated with a higher sensitivity for pain symptoms, a biased attention towards pain and a rather dysfunctional evaluation of pain (pain catastrophizing) [69, 70]. The association between headache and depression/anxiety was repeatedly confirmed in several studies [11, 71].

Life events and dysfunctional stress coping turned out to be significant predictors for the ‘high pain class’ but not for the ‘moderate decreasing pain class’ in girls. Post-hoc analyses revealed significantly higher mean values for life events as well as dysfunctional stress coping in the ‘high pain class’ as compared to the ‘moderate decreasing pain class’ (all *p* < 0.001). It may be assumed that girls of the ‘high pain class’ were more often confronted with stressful life events without being able to use functional coping strategies. This interpretation is supported by former studies [72, 73].

Because of the steady increase of headache frequency in the ‘increasing pain class’ it may be hypothesised that these children will suffer from a further increase of headache frequency during their adolescence. Thus, especially for this group, it is necessary to identify risk factors which can explain this course of symptoms. Migraine did not turn out to be a significant predictor for belonging to the ‘increasing pain class’. This may easily be explained by the non-existence of headache at first wave. Moreover, not being affected by headache may explain the irrelevance of a potential parental pain model [68]. Only if headache symptoms are relevant aspects of the child’s daily life a parental pain model can come into effect. However, internalising symptoms as well as school burden significantly increased the risk of belonging to the ‘increasing pain class’. It may be assumed that this group describes girls being especially sensitive for the beginning puberty with increased psychosocial stressors and somatic symptoms. This enhanced sensitivity may result in depressive/anxious symptoms, a biased attention towards pain and increased stress experienced in school. These variables are all associated with headache symptoms [11, 24]. However, since most of the included variables are not significantly associated with this trajectory class, further research is necessary in order to define additional risk factors in an adequate methodological manner. Referring to the assumption of a higher sensitivity for psychosocial processes and changes in this group of girls, it may be hypothesised that variables like a lack of social support, stress in the peer group or psychosocial experiences of loss (e.g., break-up with friends or romantic partners) may be relevant factors for an increase of headache in that specific group [58, 74].

For boys, again, migraine and parental headache were able to differentiate between the classes. In contrast to the girls’ subsample, depression/anxiety was not significantly associated with a pain class in the boys’ data set. Our results show higher depression/anxiety scores for girls as compared to boys. This supports former studies [9, 39]. It may be possible that the general low level of depression/anxiety in boys explains the lacking significance of this variable in the boys’ headache trajectories. However, as for girls, life events and dysfunctional stress coping turned out to be significant predictors for a pain class in the boys’ subsample. Post-hoc analyses revealed significantly higher mean values for life events (*p* = 0.005) and dysfunctional stress coping strategies (*p* < 0.001) in the ‘moderate stable pain class’ as compared to the ‘no pain class’. Just as for the girls’ subsample, this finding may also be explained by a lack of effective coping strategies when being confronted with stressful situations, i.e., life events.

In sum, our results point to the necessity of early identification of children and adolescents at risk of becoming significantly affected by headache. Additionally, for children suffering from headache already at baseline, the identification of possible factors that contribute to the maintenance of headache is crucial. In the light of our results especially migraine and parental headache seem to be risk factors for syndrome patterns in both sexes. Against the background of a parental influence, not only a possible genetic predisposition should be considered, but also the influence of dysfunctional pain-related modelling mechanisms. Additionally, inadequate coping with headache as well as depressive cognitive styles, both associated with negative self-instructions, a rather anxious-negative and biased attention towards pain, an increased sensitivity for pain, and tendency to ruminate about pain seem to heighten the probability of increased headache frequency and a corresponding pain-associated disability [70, 75].

### Strengths and limitations of the study

One of the main strengths of the current work is the large sample size and the random choice that allowed insight into paediatric headache trajectories in a population-based sample. Moreover, by using a special statistical method, conducting sex-specific analyses and including various relevant predictors, the scope in this study field could be extended. Insight into genetic, somatic and (socio-)psychological variables that need to be considered in preventive plans and treatment programs could be facilitated.

Another advantage of our study is the self-report that was used in order to assess paediatric headache. Several studies showed that the inclusion of the child’s perception of pain symptoms is crucial and cannot be substituted by the parental report, since there is a systematic difference between children’s and parents’ estimation, i.e., an underestimation of the child’s headache prevalence by parents [76, 77].

One limitation that needs to be considered is the assessment of headache frequency. Our question demanded the classification of headache days in relation to a specific period of time (either the last week, the last month or the last six months) depending on the precedent answer of the respondents. Especially for younger children, it may have been difficult to give a correct estimate of the number of headache days, particularly for the categories that included a greater time period. Moreover, in order to be able to extrapolate the answers of the questioned children and adolescents to the last six months, the answers were weighted correspondingly. This resulted in great intra-individual differences over the assessment points, especially for the ‘high pain class’ since this class was comprised of girls with significant intraindividual changes across time. Future studies should avoid the necessity of weighting by creating a scale for a differentiated and valid assessment of very low as well as very high headache levels, so that the valid assessment of severely affected patients can also be considered. One possible type of assessment would be the use of a pain diary. However, the effort that would be necessary (personal contact, filling out the diary over at least four weeks) would not be compatible with the type of study and the study sample (postal survey, high number of respondents, children).

The type of assessment and the chosen sample influence possible interpretations of our results. At each measurement point a cohort of a specific age range (9–14 years at wave one) was examined. Hence, we can make statements about changes in headache across the course of the study, but not particularly concerning changes across childhood or adolescence.

In our study we used migraine as a predictor and disregarded tension-type headache (TTH). This decision was mainly based on studies revealing a higher long-term psychosocial disability due to migraine in contrast to TTH [11]. However, future studies should compare the influence of the two headache types on paediatric headache and related disabilities over time. Moreover, we used the ICHD-II criteria to diagnose migraine since the classification of headache was based on the ICHD-II at the time of the conduction of the study. Future studies should rely on ICHD-III beta when diagnosing migraine. It is unmistakably evident that a clinical relevant diagnosis of headache has to rely on a comprehensive set of instruments for clinical examination of patients. Our method of analysis rather served to describe and differentiate headache in the general population.

## Conclusion

By utilising a highly complex statistical method we were able to take into account the heterogeneity of paediatric headache in a population-based sample of children and adolescents and to consider potential risk factors that are associated with unfavourable courses of headache. Our findings support prior work in that only a minority of children and adolescents suffered from recurrent headache and associated disability. As expected these groups were existent only in the girls’ subsample. In general, sex-specific analyses revealed a higher incidence of headache as well as a higher heterogeneity in girls as compared to boys. In the girls’ subsample, we identified two classes that seem to be in special need of attention. For these groups of girls, the acquisition of effective stress coping strategies together with the treatment of depressive, catastrophising and anxious cognitions should be in focus. Moreover, the diagnosis of migraine seems to be a valid indication for a higher headache frequency or a stability of headache over time, respectively. This result supports the necessity of adequately diagnosing headache types and providing effective treatment options. Concerning the relevance of parental headache, parents in pain as well as their children should be made aware of potential modelling mechanisms and be offered adequate treatment strategies for their own headache symptoms.

Future studies should analyse the potential of the extracted classes of the current study in predicting later functional, emotional, and psychosocial disability.

### Ethical statements

The ethics committee of the German Association of Psychology approved the study protocol. Data safety procedures were checked and approved by the university data protection representative. The surveyed households were informed of data protection regulations and procedure, the aims of the study, and the voluntary nature of the participation.
